# Safety of antidepressants in adults aged under 65: protocol for a cohort study using a large primary care database

**DOI:** 10.1186/1471-244X-13-135

**Published:** 2013-05-10

**Authors:** Carol Coupland, Richard Morriss, Antony Arthur, Michael Moore, Trevor Hill, Julia Hippisley-Cox

**Affiliations:** 1 Division of Primary Care, University of Nottingham, 13th floor, Tower Building, University Park, Nottingham NG7 2RD, UK; 2 Division of Psychiatry, University of Nottingham, Nottingham, UK; 3 School of Nursing Sciences, University of East Anglia, Norwich, UK; 4 Primary Care Research Network South West, University of Southampton, Southampton, UK

**Keywords:** Antidepressants, Depression, Cohort, Safety

## Abstract

**Background:**

Antidepressants are among the most commonly prescribed drugs in primary care in England and their use is increasing. This is largely due to longer durations of treatment of depression. Observational studies have shown some differences in adverse outcomes associated with different antidepressant drugs but relatively little is known about their relative safety particularly with long term use. The primary aim of this study is to determine the relative and absolute risks of pre-defined adverse events comparing different classes of antidepressant drugs in adults aged under 65 years and diagnosed with depression.

**Methods/design:**

The study will identify a cohort of patients with a first recorded diagnosis of depression between 1/1/2000 and 31/07/2011, and made between the ages of 20 to 64 years using a large primary care database (QResearch). Patients will be followed up until 1/08/2012. Details of all prescriptions for antidepressants in patients in the cohort will be extracted, including the date of each prescription, the type of antidepressant drug, the dose and total quantity prescribed. Prospectively recorded data will be used to ascertain information on adverse outcomes that occurred during follow-up and after entry into the cohort. These are: all-cause mortality, suicide, attempted suicide/self-harm, sudden death, antidepressant overdose/poisoning, myocardial infarction, stroke/transient ischaemic attack, cardiac arrhythmia, epilepsy/seizures, upper gastrointestinal bleeding, falls, fractures, adverse drug reactions and motor vehicle crashes. Cox proportional hazard models will be used to estimate the association of the outcomes with class of antidepressant drug adjusting for potential confounding variables. The analyses will also examine associations by duration and dose and with the most frequently prescribed individual antidepressant drugs. Self-controlled case series analyses will be used to estimate the relative incidence of the outcomes of interest for defined time periods of antidepressant use.

**Discussion:**

The results of this study will help to establish the relative safety and balance of risks for different antidepressant drugs in people aged under 65.

## Background

Antidepressant drugs are among the most commonly prescribed drugs in primary care; more than 46 million prescriptions for antidepressants were issued in England in 2011, an increase of 48% compared with 2006 [1]. The majority of antidepressant prescriptions in 2012 (54%) were for selective serotonin reuptake inhibitors (SSRIs) with 29% for tricyclic antidepressants (TCAs) and 17% for other antidepressant drugs [2]. Studies have shown that the large rise in antidepressant volumes is mainly due to longer durations of treatment [3,4]. A systematic review of antidepressants compared with placebo in trials conducted in primary care in patients aged less than 65 found that both TCAs and SSRIs are effective treatments for depression in primary care [5]. Most trials of antidepressants are however short term, in selected populations and comparatively little is known about their relative safety particularly with long term use.

Some observational studies have shown increased risks of suicide associated with antidepressant use [6-8], with differential effects according to age [8], but there is little evidence to support any differences between classes of antidepressant. Other adverse effects associated with antidepressants in some observational studies include myocardial infarction [9,10], stroke [11-14], fracture [15,16], and gastrointestinal bleeding [17], but findings have been inconsistent, many studies have been small and short-term, some studies have only studied one class of antidepressants, and few studies have looked at individual drugs or dose, so the evidence on antidepressant safety is far from conclusive.

A study we carried out to investigate safety and harms of antidepressant drugs in older people aged 65 years and over [13,18] found that selective serotonin reuptake inhibitors and drugs in the group of other antidepressants were associated with an increased risk of several adverse outcomes compared with tricyclic antidepressants. The current study in adults aged less than 65 years will compare results in younger people so that a comprehensive assessment can be made of antidepressant safety across adults of all ages. The primary study objective is to determine the relative and absolute risks of pre-defined adverse events in adults aged under 65 and diagnosed with depression comparing the different classes of antidepressant drugs.

## Methods/design

The study will use a large primary care database (QResearch, version 34) to identify an open cohort of patients with a first recorded diagnosis of depression between 1/1/2000 and 31/07/2011, and made between the ages of 20 to 64. QResearch is a large consolidated database derived from the anonymised health records of over 12 million patients. The data currently come from more than 600 general practices across the UK using the EMIS clinical computer system.

### Eligibility criteria

Diagnostic Read codes will be used to identify an open cohort of patients with a diagnosis of depression, using case definitions that have been used in previous studies [7,9,18]. Patients will be eligible for inclusion in the study cohort if they have a first recorded diagnosis of depression, made between the ages of 20 and 64 and between 1/1/2000 and 31/07/2011. Patients will only be eligible for inclusion if their diagnosis occurred at least 12 months after registration with a study practice and after the date of the installation of the practice EMIS computer system The cohort of eligible patients will include patients with a diagnosis of depression who were treated with antidepressant medication and also patients with a diagnosis of depression who did not receive antidepressant treatment. To reduce the risk of indication and channeling bias patients who received prescriptions for antidepressants but did not have a recorded diagnosis of depression will not be eligible for inclusion as the prescriptions may have been for conditions other than depression, such as neuropathic pain or eating disorders.

### Exclusion criteria

Patients will be excluded from the cohort if they: were temporary residents; had a diagnosis of schizophrenia, bipolar disorder or other types of psychoses or had been prescribed lithium or antimanic drugs at the study entry date; had a previous recorded diagnosis of depression; or had received prescriptions for an antidepressant either before the study start date (1/1/2000), before their date of registration with the practice (if later), before the age of 20 or more than 36 months before their recorded diagnosis of depression.

### Duration of follow up

Since some patients may have received an antidepressant for treatment of depression before the diagnosis of depression was recorded, the index date which marks the date of entry into the study cohort for analysis will be defined as the date of the first recorded diagnosis of depression, or the date of the first prescription for an antidepressant if that occurred before the recorded date of depression.

Participants will be followed-up in the database from their index date until the earliest of: date of death, date of leaving the practice, or the end of the follow-up period (1/08/2012).

### Exposures

The exposure of interest is antidepressant medication. We will extract details of all prescriptions for antidepressants in patients in the study cohort, following their initial diagnosis of depression (or first prescription for an antidepressant if no more than 36 months before their recorded diagnosis of depression), up to the study end date (1/08/2012), or the date of death or leaving the practice if this occurs earlier. Extracted information will include the date of each prescription, the type of antidepressant drug, the dose and total quantity prescribed.

The antidepressant drugs will be grouped for analysis according to the major classes as described in the British National Formulary (BNF), namely:

•tricyclic and related antidepressants (TCAs - section 4.3.1),

•selective serotonin reuptake inhibitors (SSRIs - section 4.3.3),

•monoamine oxidase inhibitors (MAOIs - section 4.3.2)

•other antidepressants (Others - section 4.3.4).

Some patients may receive prescriptions for different drugs within a class or drugs from different classes on the same date. These prescriptions will be identified and classified as combined prescriptions for some analyses.

The number of prescriptions, duration of treatment and dose of the antidepressant drugs will be examined in the analyses. We will determine the duration of each prescription in days by dividing the number of tablets prescribed by the dosing directions (e.g. number of tablets to be taken per day). Where the number of tablets prescribed is recorded but the dosing directions are missing or not sufficiently detailed we will use an assumed duration based on the median duration of prescriptions for those prescriptions where dosing directions were available, taking account of the number of tablets prescribed as in our previous study [18].

We will calculate the daily dose of each prescription by multiplying the specified dose of each tablet prescribed by the number of tablets to be taken each day. To enable comparison of doses between the antidepressant classes, we will convert the dose per day for each prescribed drug to a defined daily dose (DDD), defined as the assumed average maintenance dose per day for a drug used for its main indication in adults, using the DDD values assigned by the World Health Organisation’s Collaborating Centre for Drug Statistics Methodology (http://www.whocc.no/atc_ddd_index).

Individual antidepressant drugs will also be assessed where numbers are sufficient. In our previous study we examined the top 11 most commonly prescribed individual antidepressant drugs separately in the analyses of the more common outcomes [18].

### Outcomes

We have identified a number of potential adverse effects of antidepressants from the literature. Information on these outcomes will be extracted from the primary care computer records of patients in the cohort and from linked death certificates for patients who died during the study period. Outcomes will only be included if they occurred after the index date of entry into the study cohort. Computer recorded Read codes and ICD 9 and 10 codes from linked death certificates where appropriate will be used to identify patients with each of the outcomes of interest.

The outcomes that will be assessed are:

•all-cause mortality

•suicide (including open verdicts)

•attempted suicide/self-harm

•sudden death

•overdose/poisoning with an antidepressant

•myocardial infarction

•stroke/transient ischaemic attack (TIA)

•cardiac arrhythmia

•epilepsy/seizures

•upper gastrointestinal bleeding

•falls

•fractures

•adverse drug reactions (including bullous eruption)

•motor vehicle crash.

The date of occurrence of the outcome used in analysis will be the first recorded date of the outcome during follow-up.

### Confounding variables

Information will also be extracted on potential confounding variables including:

•age at index date

•sex

•year of diagnosis of depression

•severity of index diagnosis of depression (categorised as mild, moderate or severe based on the Read code for the index diagnosis, using codes published by Martinez and colleagues [7] and some additional classification by a member of the study team (RM))

•deprivation, based on Townsend deprivation score for the patients postcode in quintiles

•smoking status (non-smoker, ex-smoker, current smoker – light/moderate/heavy)

•alcohol intake (none, trivial <1 unit/day, light 1–2 units/day, medium 3–6 units/day, heavy 7–9 units/day, very heavy >9 units/day)

•ethnic group (white/not recorded, Indian, Pakistani, Bangladeshi, other Asian, black African, black Caribbean, Chinese, other including mixed)

•comorbidities at baseline (coronary heart disease, diabetes, hypertension, stroke/TIA, cancer, epilepsy/seizures, hypothyroidism, rheumatoid arthritis, osteoarthritis, osteoporosis, liver disease, renal disease, asthma/chronic obstructive airways disease, obsessive-compulsive disorder) identified using appropriate Read codes in the patients records

•use of other drugs at baseline (statins, NSAIDS, anti-psychotics, aspirin, antihypertensive drugs, anticonvulsants, hypnotics/anxiolytics, oral contraceptives, HRT, bisphosphonates warfarin and anticoagulants).

•In addition for the analysis of suicide as an outcome, previous attempted suicide/self-harm at baseline will be considered a confounding variable. For the analysis of fracture, previous falls will be considered a confounding variable.

### Statistical analysis

Descriptive analyses will be carried out to describe baseline characteristics of patients in the study cohort. We will describe patterns of antidepressant use according to class and individual type of antidepressant prescribed, duration of use and dose, and will examine these patterns by sex, age group, deprivation, ethnic group, use of other medications, comorbidities and study year.

The primary statistical analysis for the cohort study will comprise a series of survival analyses to assess the relationship between exposure to antidepressant drugs and the adverse outcomes. Cox’s proportion hazards models will be used to analyse the cohort data to calculate hazard ratios and 95% confidence intervals, with antidepressant exposure treated as a time-varying exposure. The time varying analysis accounts for patients starting and stopping treatment during follow-up and also changing between treatments during follow-up. For example, a patient diagnosed with depression who received treatment with an SSRI 3 months after diagnosis and who stopped treatment 9 months after diagnosis will be analysed in the “no current treatment” group for time 0 to 3 months, the “SSRI treatment” group for 3 to 9 months, and the “no current treatment” group after 9 months

The main statistical analysis will only include adverse outcomes recorded within the first 5 years after the index date (because the confounders may change considerably over a longer period). The entry date into the analysis will be the index date (earliest of first diagnosis of depression or first antidepressant prescription), and the outcome date will be the earliest of: the date of diagnosis of the outcome of interest, or the date of death if the outcome was only recorded on their death certificate. We will use the first recorded diagnosis of the outcome of interest rather than recurrent events. Patients who do not have the outcome of interest within 5 years will be censored at the earliest of: date of death, date of leaving the practice, date of the latest download of data, the study end date or 5 years after the index date. For the analysis of each outcome we will exclude patients who had already had the outcome at baseline.

For the main analyses patients will be considered to be exposed to a drug throughout periods covered by the duration of the prescriptions if there were no gaps of more than 90 days between the end of one prescription and the start of the next prescription to allow for not having a precise date when the patient finished the prescription. A prescription after more than 90 days will count as a new treatment episode. Where there are gaps of more than 90 days between the end of one prescription and the start of the next prescription then patients will be counted as exposed to antidepressant medication for the first 90 days of the gap and then unexposed until the date of the next prescription.

The analysis will calculate hazard ratios for current treatment for each separate class of antidepressants (SSRIs, TCAs and other antidepressants) compared with no current treatment. It is anticipated that the number of patients prescribed MAOIs will be too small for this group to be analysed, so any patients prescribed MAOIs at any time will be excluded from the analysis. Further analyses will compare each antidepressant class directly with SSRIs as the reference category, and will assess associations with dose of antidepressant according to each antidepressant class and duration of antidepressant use treated as a time-varying exposure (categorised for each class of antidepressant drug as: 1–28 days after the first prescription in a treatment episode; 29–84 days after and 85+ days after) and time since stopping (1–28 days, 29 to 84 days and 85 to 182 days after stopping treatment). Analyses will be carried out for individual antidepressants where numbers are sufficient. We will carry out unadjusted and adjusted analysis controlling for the potential confounding variables listed above.

For the more common outcomes we will carry out subgroup analyses and tests of interaction to examine whether associations differ by age, sex and ethnic group. The assumptions of the Cox proportional hazards model will be checked graphically.

We will use a P value of < 0.01 (two tailed) to determine statistical significance since there are multiple outcomes. We will carry out Wald’s significance tests to determine whether there are significant differences between the antidepressant classes overall, excluding the group with no current treatment. We will also do this for the analyses of individual drugs.

As a sensitivity analysis we will repeat the analyses above restricted to patients who received at least one prescription for an antidepressant during follow-up, since the untreated group may differ in a number of ways which could lead to residual confounding. A further analysis will include all adverse outcomes that occur within the entire follow-up period, so associations with long term use can be examined.

Absolute risks of the adverse events will also be estimated and presented. We will estimate absolute risks of the adverse events at 1, 3 and 5 years from the baseline date according to each antidepressant class and for individual drugs using a published formula [19].

### Sample size

All eligible patients on the database will be included in the study to maximise study power.

### Self-controlled case series analysis

We will also perform self-controlled case series analyses using data only on patients who have had adverse events. This is an internally controlled method whereby analyses are carried out only in patients with the outcome of interest, which has the advantage of implicitly adjusting for all measured and unmeasured fixed confounding variables within patients and so reduces residual confounding and indication bias [20,21].

We will use conditional Poisson regression to estimate the relative incidence of the outcomes of interest for defined time periods of risk after the first prescription for antidepressants compared with an unexposed baseline period. We will only use the first recorded diagnosis of the outcome of interest rather than recurrent events. Patients who have the outcome of interest occurring on the same day as their first prescription for antidepressants will be distinguished in the analysis. We will account for multiple periods of exposure in the analysis, defining a period of antidepressant treatment as one without gaps of more than 90 days between the end of a prescription and the start of the next prescription. A prescription after more than 90 days will count as a new treatment episode. We will adjust for age in the analyses.

The time periods for assessing potential short term effects of antidepressants will be defined for each class of antidepressant drug as: 0 days (day of first prescription in each treatment episode); 1–28 days after the first prescription; 29–84 days and 85+ days (remaining treatment period); and periods after stopping treatment (1–28 days, 29 to 84 days and 85 to 182 days after stopping). These risk periods were selected as they enable examination of short term and longer term effects of antidepressants on the risks of adverse events and are the same as those used in our previous study of antidepressants in older people [13]. The 28 days before the first prescription in each treatment episode will be considered as a separate category, as an occurrence of the outcome of interest in this period could affect the probability of an antidepressant prescription. Time periods outside these specified risk periods will contribute to the baseline person time i.e. the unexposed periods.

The baseline period will be restricted to the year before the index date (earliest of first diagnosis of depression or first antidepressant prescription) and up to 5 years after diagnosis, to reduce the influence of time varying factors.

## Discussion

The findings of this study will help to establish the relative safety and balance of risks for different antidepressant drugs in people aged under 65 years, in order to support decision making for clinicians and patients considering treatment with antidepressant drugs.

Primary care databases with their large volumes of high quality data on representative populations over many years are well suited to the study of unintended effects of medication.

The current study has a number of strengths. It will include a large and representative cohort of people with a recorded diagnosis of depression and will be used to calculate absolute as well as relative risks of a number of serious adverse outcomes. The data on patients in the cohort were recorded prospectively, so all information on prescriptions for antidepressants and potential confounding variables was recorded before the occurrence of an adverse outcome, meaning that results will not be susceptible to recall bias.

Prescriptions are reliably recorded in primary care records, and the drugs under consideration in this study are only available on prescription reducing misclassification of exposure. We will have detailed information on prescriptions for antidepressants issued in primary care throughout the follow-up period, so we will be able to carry out comprehensive analyses investigating associations by dose and duration and for individual drugs as well as for antidepressant class. This contrasts with many cohort or case–control studies in which information on antidepressant use is self-reported or collected only at the start of the study.

The main concern with observational studies of drug safety such as this one is indication bias which occurs when patients are prescribed drugs for a condition that is itself associated with the outcome of interest. To reduce this bias, we will restrict our study cohort to only include patients with a recorded diagnosis of depression, so that all patients had the same indication for treatment. We will also adjust for a range of potential confounding factors which would be expected to reduce the effect of this bias

Residual confounding may also affect the findings, as certain potential confounding variables may not be recorded on the database or may not be recorded in sufficient detail to completely remove their confounding effect. The self-controlled case series analysis will help to determine the extent of this, since it is a within patients comparison, which implicitly removes the effects of all characteristics that vary between patients, irrespective of whether or in how much detail they have been recorded on the database, assuming that they do not vary over time within the observation period [20,21].

### Ethical arrangements

The project has been independently peer reviewed and accepted by the QResearch Scientific board and has been reported to Trent Research Ethics Committee in accordance with the agreed procedure with the Committee (reference No MREC/03/4/021).

## Competing interests

Julia Hippisley-Cox is director of QResearch which is a not for profit venture between the University of Nottingham and EMIS (commercial supplier of GP clinical systems).

Carol Coupland, Richard Morriss, Tony Arthur, Trevor Hill and Michael Moore declare that they have no competing interests.

## Authors’ contributions

CC, JHC, RM, TA, and MM contributed to the overall design and conception of the study. CC wrote the first draft of this manuscript. TH contributed to the statistical aspects of the study. All authors contributed to drafting of this manuscript. All authors read and approved the final manuscript.

## Pre-publication history

The pre-publication history for this paper can be accessed here:

http://www.biomedcentral.com/1471-244X/13/135/prepub
